# Thermodynamic properties of microorganisms: determination and analysis of enthalpy, entropy, and Gibbs free energy of biomass, cells and colonies of 32 microorganism species

**DOI:** 10.1016/j.heliyon.2019.e01950

**Published:** 2019-06-18

**Authors:** Marko Popovic

**Affiliations:** Biothermodynamics, TUM School of Life Sciences Weihenstephan, Technical University of Munich, Maximus-von-Imhof-Forum 2, 85354, Freising, Germany

**Keywords:** Chemical Engineering, Thermodynamics, Physical chemistry

## Abstract

Thermodynamic analysis is an important part of chemical engineering. However, its application in biotechnology has been hampered by lack of data on thermodynamic properties of microorganism biomass. In this paper, a review was made of methods for estimation of thermodynamic properties of biomass, including standard enthalpy of combustion *h*_*C*_*⁰*, enthalpy of formation *h*_*f*_*⁰*, entropy *s⁰*, and Gibbs free energy of formation *g*_*f*_*⁰*. These parameters were calculated on molar and mass specific basis for 32 microorganism species, including 14 bacteria, 7 yeast and 11 algae species. It was found that *h*_*f*_*⁰*, *s⁰*, *g*_*f*_*⁰* are, respectively, similar for all the analyzed species, due to the fact that all living organisms have a common ancestor and thus a similar chemical composition. Furthermore, all the analyzed microorganisms have negative *h*_*f*_*⁰*, originating from partial oxidation of all other elements by oxygen and nitrogen. A brief review was given of microorganism endogenous and growth metabolic rates. Finally, based on the determined thermodynamic properties, entropy of individual *E. coli* and *Pseudomonas* cells were determined and entropy of a *Pseudomonas* colony during its lifespan was calculated and analyzed. Three periods can be distinguished in the existence of a microorganism colony: (a) accumulation period when cell number, mass and entropy increase, (b) steady state period when they are approximately constant, and (c) decumulation period when they decrease.

## 1 Introduction

Animate matter represents a highly organized, self-assembled amount of substance clearly separated by a semipermeable membrane from its environment (inanimate matter) (Morowitz, 1992). In biological terms, an organism is characterized by cellular structure that corresponds to a thermodynamic system (Morowitz, 1955; Schrödinger, 2003; von Bertalanffy, 1950; Balmer, 2010; Popovic, 2017a, 2018, Demirel, 2014). Animate matter represents an open system (von Bertalanffy, 1950). Moreover, the seven fundamental characteristics of life are: (1) ordered cell structure, (2) reproduction, (3) growth and development, (4) energy utilization, (5) response to the environment, (6) homeostasis, and (7) evolutionary adaptation (Campbell and Reece, 2002). Thus, an organism also represents a growing open system due to accumulation of matter and energy (Popovic, 2017b). Thus, animate matter performs thermodynamic processes corresponding to biological (life) processes.

Thermodynamics is widely applied in chemical engineering, but its full potential is still unexploited in biotechnology. Thermodynamics has played a fundamental role in the development of chemical industry, facilitating design of new technologies, enabling optimization of existing processes and avoiding difficult measurements. Therefore, there are three kinds of information that one must have for chemical engineering: balances, kinetics and thermodynamics. However, in biotechnology, a new and rapidly developing field of engineering, there is still a lack of information for thermodynamic analysis, forcing process development to be based on expensive measurements and making their optimization difficult (von Stockar, 2010). Therefore, it would be beneficial to have better means to predict thermodynamics of biotechnological processes.

Many biotechnological processes utilize growth of microorganisms, which can be represented by a chemical reaction, also known as growth reaction (von Stockar, 2010). Growth reactions, like all other reactions, have thermodynamic parameters that can be calculated as the difference of products and reactants. Since a major product in microorganism growth are new microorganisms, to find reaction thermodynamics, it is necessary to have standard thermodynamic properties of biomass.

The goal of this paper is to determine standard thermodynamic parameters of microorganism biomass and based on them make an analysis of microorganism growth. Section 2 reviews the methods used for determining biomass thermodynamic parameters, including enthalpy of combustion, enthalpy of formation from elements, entropy and Gibbs free energy of formation from elements. Section 3 gives the results and indicates general trends. Finally, section 4 gives explanations for the trends in the results, discusses microorganism metabolic rates and applies the results to growth of a *Pseudomonas* colony. Entropy of a *Pseudomonas* cell is calculated, and entropy change during growth of the entire colony is presented and discussed.

## 2 Methods

This section reviews methods to estimate thermodynamic parameters of microorganism biomass. First, enthalpy of combustion estimation methods are reviewed and compared to experimental microorganism biomass combustion data. Based on the determined enthalpies of combustion, a method is shown how to find enthalpies of formation of microorganisms. The discussion then turns to estimating entropy of dry microorganism biomass from its elemental composition, using the Battley equation.

Animate matter (a living organism) consists of water and dry biomass. The methods reviewed in this section are used to find thermodynamic properties of dry microorganism biomass. Once these are known, they are added to corresponding thermodynamic properties of water to find thermodynamic properties of animate matter, that is of the living organism (section 4).

Empirical formulas have been collected from the literature for 32 microorganism species, including 14 bacteria, 7 yeast and 11 algae species. They are given in Tables 1 and 2. From Tables 1 and 2, it can be seen that the most abundant elements in all microorganisms are C, H, O and N, which can be used to roughly represent their composition. Thus, the average chemical formula of dry bacterial biomass was found from data in Table 1 to be CH_1.7_O_0.4_N_0.2_, for fungi it is CH_1.7_O_0.5_N_0.1_ and for algae CH_1.7_O_0.5_N_0.1_. All other elements are present in amounts a magnitude lower or less.

**Table 1:** Standard enthalpy of formation ${\bar{h}}_{f}^{o}$, entropy ${\bar{s}}^{o}$, and Gibbs free energy of formation ${\bar{g}}_{f}^{o}$ normalized per mole of carbon atoms (UCF formula) of dry bacteria, fungi and algae biomass. To appreciate the size of a cell, a single *Magnetospirillum gryphiswaldense* cell can be described by C_2.31 × 10_^10^H_4.68 × 10_^10^O_2.93 × 10⁹_N_6.56 × 10_^9^^9^Fe_4.00 × 10⁷_. The thermodynamic parameters for entire cells are given by the equation *X*⁰_*cell*_ = *n*_*bio*_ · ${\bar{x}}_{bio}^{o}$ + *n*_*w*_ · ${\bar{x}}_{w}^{o}$ where *X*⁰_*cell*_ is thermodynamic parameter (*H*_*f*_, *S*, or *G*_*f*_) for a single cell, ${\bar{x}}_{bio}^{o}$ thermodynamic parameter for biomass from this table, *n*_*bio*_ number of moles of dry matter, ${\bar{x}}_{w}^{o}$ thermodynamic parameter for water and *n*_*w*_ number of moles of water in the cell (Section 4.3).

Name	Formula	Reference	${\bar{h}}_{f}^{o}$ (kJ/mol)	${\bar{s}}^{o}$ (J/mol K)	${\bar{g}}_{f}^{o}$ (kJ/mol)
*BACTERIA*					
Bacteria (general)	CH_1.666_O_0.270_N_0.200_	Abbott and Clamen (1973)	−61.90 ± 3.32	30.15 ± 5.94	− 22.82 ± 5.09
Aerobacter aerogenes	CH_1.830_O_0.550_N_0.250_	Naresh et al. (2011)	− 129.35 ± 6.93	38.42 ± 7.57	− 79.55 ± 9.19
Brevibacterium flavum	CH_1.80_O_0.33_N_0.19_	Duboc et al. (1999), Table 9	− 79.50 ± 4.26	32.76 ± 6.45	− 37.04 ± 6.19
Bacillus cereus	CH_1.49_O_0.43_N_0.22_	Duboc et al. (1999), Table 9	− 91.88 ± 4.92	31.43 ± 6.19	− 51.14 ± 6.77
Corynebacterium glutamicum	CH_1.78_O_0.44_N_0.24_	Duboc et al. (1999), Table 9	− 103.31 ± 5.54	35.52 ± 7.00	− 57.27 ± 7.62
Escherichia coli	CH_1.770_O_0.490_N_0.240_	Bauer and Ziv (1976)	− 114.11 ± 6.12	36.36 ± 7.16	− 66.98 ± 8.25
E. coli	CH_1.74_O_0.34_N_0.22_	Duboc et al. (1999), Table 9	−79.81 ± 4.28	32.75 ± 6.45	−37.36 ± 6.20
E. coli K-12: grown on Acetic acid	CH_1.54_O_0.4_N_0.21_	Battley (1992), Table 3	−86.80 ± 4.65	31.28 ± 6.16	−46.25 ± 6.49
E. coli K-12: grown on glucose	CH_1.74_O_0.464_N_0.26_	Battley (1992), Table 3	−107.38 ± 5.76	35.85 ± 7.06	−60.91 ± 7.86
E. coli K-12: grown on glucose	CH_1.81_O_0.40_N_0.22_	Battley (1992), Table 3	−95.37 ± 5.11	34.76 ± 6.85	−50.32 ± 7.15
E. coli K-12: grown on glucose	CH_1.73_O_0.53_N_0.235_	Battley (1992), Table 3	−121.73 ± 6.52	36.54 ± 7.20	−74.36 ± 8.67
E. coli K-12: grown on glucose	CH_1.78_O_0.511_N_0.237_	Battley (1992), Table 3	−119.09 ± 6.38	36.83 ± 7.25	−71.36 ± 8.55
E. coli K-12: grown on glucose	CH_1.81_O_0.49_N_0.234_	Battley (1992), Table 3	−115.38 ± 6.18	36.74 ± 7.24	−67.76 ± 8.34
E. coli K-12: grown on glucose	CH_1.54_O_0.34_N_0.24_	Battley (1992), Table 3	−73.46 ± 3.94	30.67 ± 6.04	−33.71 ± 5.74
E. coli K-12: grown on Succinic acid	CH_1.56_O_0.36_N_0.23_	Battley (1992), Table 3	−78.54 ± 4.21	31.12 ± 6.13	−38.20 ± 6.04
E. coli W: grown on glucose	CH_1.698_O_0.427_N_0.250_	Battley (1992), Table 3	−97.82 ± 5.24	34.45 ± 6.79	−53.17 ± 7.27
E. coli W: grown on glycerol	CH_1.698_O_0.427_N_0.250_	Battley (1992), Table 3	−97.82 ± 5.24	34.45 ± 6.79	−53.17 ± 7.27
Flavobacterium dehydrogenans	CH_1.63_O_0.40_N_0.21_	Duboc et al. (1999), Table 9	−89.66 ± 4.81	32.38 ± 6.38	−47.68 ± 6.71
Klebsiella aerogenes	CH_1.750_O_0.430_N_0.220_	Naresh et al. (2011)	−100.14 ± 5.37	34.60 ± 6.82	−55.28 ± 7.40
Klebsiella aerogenes	CH_1.730_O_0.430_N_0.240_	Naresh et al. (2011)	−99.50 ± 5.33	34.72 ± 6.84	−54.50 ± 7.37
Klebsiella aerogenes	CH_1.750_O_0.470_N_0.170_	Naresh et al. (2011)	−109.03 ± 5.84	34.47 ± 6.79	−64.34 ± 7.87
Klebsiella aerogenes	CH_1.730_O_0.430_N_0.240_	Naresh et al. (2011)	−99.50 ± 5.33	34.72 ± 6.84	−54.50 ± 7.37
Lactobacillus helveticus	CH_1.58_O_0.39_N_0.23_	Duboc et al. (1999), Table 9	−85.84 ± 4.60	31.94 ± 6.29	−44.45 ± 6.48
Magnetospirillum gryphiswaldense	CH_2.060_O_0.130_N_0.280_Fe_0.00174_	Naresh et al. (2011)	−44.02 ± 2.36	33.72 ± 6.64	−0.31 ± 4.34
Methanobacterium thermoautotrophicum	CH_1.63_O_0.43_N_0.22_	Duboc et al. (1999), Table 9	−96.32 ± 5.16	33.14 ± 6.53	−53.37 ± 7.11
Methylococcus capsulatus	CH_2.000_O_0.500_N_0.270_	van Dijken and Harder (1975)	−123.64 ± 6.63	39.90 ± 7.86	−71.93 ± 8.97
Paracoccus denitrificans	CH_1.510_O_0.460_N_0.190_	Shimizu et al. (1978)	−99.18 ± 5.32	31.71 ± 6.25	−58.08 ± 7.18
Paracoccus denitrificans	CH_1.810_O_0.510_N_0.200_	Stouthamer (1977)	−119.83 ± 6.42	36.51 ± 7.19	−72.50 ± 8.57
Pseudomonas C12B	CH_2.000_O_0.520_N_0.230_	Mayberry et al. (1968)	−128.09 ± 6.87	39.56 ± 7.79	−76.80 ± 9.19
Saccharopolyspora erythraea (a)	CH_1.61_O_0.47_N_0.19_	Duboc et al. (1999), Table 9	−104.58 ± 5.61	33.12 ± 6.52	−61.65 ± 7.55
Saccharopolyspora erythraea (b)	CH_1.68_O_0.47_N_0.16_	Duboc et al. (1999), Table 9	−106.80 ± 5.72	33.44 ± 6.59	−63.46 ± 7.69
*YEAST*					
Saccharomyces cerevisiae	CH_1.613_O_0.557_N_0.158_P_0.012_S_0.003_K_0.022_Mg_0.003_Ca_0.001_	Battley, 1999a,b	−131.99 ± 7.07	34.66 ± 6.83	−87.07 9.11
Saccharomyces cerevisiae	CH_1.640_O_0.520_N_0.160_	Harrison (1967)	−116.65 ± 6.25	33.91 ± 6.68	−72.69 ± 8.24
Saccharomyces cerevisiae	CH_1.830_O_0.560_N_0.170_	Kok and Roels (1980)	−131.58 ± 7.05	37.18 ± 7.32	−83.38 ± 9.24
Saccharomyces cerevisiae	CH_1.810_O_0.510_N_0.170_	Wang et al. (1976)	−119.83 ± 6.42	35.97 ± 7.09	−73.19 ± 8.54
Saccharomyces cerevisiae Whi 2+	CH_1.65_O_0.57_N_0.14_	Duboc et al. (1999), Table 9	−128.08 ± 6.86	34.63 ± 6.82	−83.19 ± 8.90
Saccharomyces cerevisiae Whi 2−	CH_1.64_O_0.5_N_0.18_	Duboc et al. (1999), Table 9	−112.20 ± 6.01	33.88 ± 6.68	−68.28 ± 8.00
Saccharomyces cerevisiae CBS 426a	CH_1.56_O_0.52_N_0.16_	Duboc et al. (1999), Table 9	−114.10 ± 6.12	32.93 ± 6.49	−71.42 ± 8.05
Saccharomyces cerevisiae CBS 426b	CH_1.52_O_0.51_N_0.19_	Duboc et al. (1999), Table 9	−110.61 ± 5.93	32.79 ± 6.46	−68.11 ± 7.85
Candida utilis	CH_1.830_O_0.540_N_0.100_	Herbert (1976)	−127.13 ± 6.81	35.54 ± 7.00	−81.06 ± 8.90
Candida utilis	CH_1.870_O_0.560_N_0.200_	Naresh et al. (2011)	−132.85 ± 7.12	38.20 ± 7.53	−83.32 ± 9.36
Candida utilis	CH_1.830_O_0.460_N_0.190_	Naresh et al. (2011)	−109.35 ± 5.86	35.62 ± 7.02	−63.18 ± 7.95
Candida utilis	CH_1.870_O_0.560_N_0.200_	Naresh et al. (2011)	−132.85 ± 7.12	38.20 ± 7.53	−83.32 ± 9.36
Candida utilis ATCC 9950	CH_1.66_O_0.56_N_0.07_	Duboc et al. (1999), Table 9	−126.17 ± 6.76	33.31 ± 6.56	−83.00 ± 8.72
Candida kefyr NCYC 1441	CH_1.66_O_0.44_N_0.12_	Duboc et al. (1999), Table 9	−99.50 ± 5.33	31.90 ± 6.28	−58.15 ± 7.21
Debaryomyces hansenii	CH_1.71_O_0.6_N_0.10_	Duboc et al. (1999), Table 9	−136.65 ± 7.32	35.22 ± 6.94	−90.99 ± 9.39
Debaryomyces nepaliensis CBS 5921	CH_1.77_O_0.63_N_0.09_	Duboc et al. (1999), Table 9	−145.23 ± 7.78	36.35 ± 7.16	−98.10 ± 9.92
Kluyveromyces marxianus NRRL 665	CH_1.73_O_0.53_N_0.17_	Duboc et al. (1999), Table 9	−121.73 ± 6.52	35.38 ± 6.97	−75.87 ± 8.60
Zygosaccharomyces bailii NCYC 563	CH_1.63_O_0.55_N_0.13_	Duboc et al. (1999), Table 9	−123.00 ± 6.59	33.83 ± 6.66	−79.15 ± 8.58
*FILAMENTOUS FUNGI*					
Aspergillus niger	CH_1.60_O_0.55_N_0.10_	Duboc et al. (1999), Table 9	−122.04 ± 6.54	32.92 ± 6.49	−79.37 ± 8.48
Aspergillus niger (spores)	CH_1.50_O_0.53_N_0.12_	Duboc et al. (1999), Table 9	−114.42 ± 6.13	31.67 ± 6.24	−73.36 7.99
Mucor rouxii	CH_1.79_O_0.43_N_0.07_	Duboc et al. (1999), Table 9	−101.41 ± 5.44	32.40 ± 6.38	−59.40 ± 7.34
Neurospora crassa	CH_1.80_O_0.45_N_0.13_	Duboc et al. (1999), Table 9	−106.17 ± 5.69	33.98 ± 6.69	−62.12 ± 7.69
Penicillium chrysogenum	CH_1.87_O_0.22_N_0.08_	Duboc et al. (1999), Table 9	−57.27 ± 3.07	29.53 ± 5.82	−18.99 ± 4.80
*ALGAE*					
Algae (general)	CH_2.481_O_1.038_N_0.151_P_0.00943_	Wang et al. (2017)	−260.31 ± 13.95	54.03 ± 10.64	−190.27 ± 17.13
Chlamydomonas	CH_1.65_O_0.39_N_0.12_	Duboc et al. (1999), Table 9	−88.07 ± 4.72	30.82 ± 6.07	−48.12 ± 6.53
Chlorella	CH_1.719_O_0.404_N_0.175_P_0.0105_	Manahan and Manahan (2009)	−95.34 ± 5.11	33.00 ± 6.50	−52.56 7.05
Chlorella a sp. MP-1	CH_1.793_O_0.608_N_0.121_	Phukan et al. (2011)	−141.17 ± 7.57	36.78 ± 7.25	−93.49 ± 9.73
Chlorella minutissima	CH_1.714_O_0.286_N_0.143_	Prajapati et al. (2014)	−66.93 ± 3.59	30.02 ± 5.91	−28.02 ± 5.35
Chlorella pyrenoidosa	CH_1.625_O_0.250_N_0.125_	Prajapati et al. (2014)	−56.15 ± 3.01	27.92 ± 5.50	−19.96 ± 4.65
Chlorella vulgaris	CH_1.667_O_0.222_N_0.111_	Prajapati et al. (2014)	−51.30 ± 2.75	27.65 ± 5.45	−15.47 ± 4.37
Chrorella sp. ATCC 7516 (medium 5)	CH_1.76_O_0.35_N_0.09_	Duboc et al. (1999), Table 9	−82.67 ± 4.43	30.86 ± 6.08	−42.67 ± 6.24
Chlorella Spain sp. ATCC 7516 (medium S)	CH_1.78_O_0.36_N_0.12_	Duboc et al. (1999), Table 9	−85.53 ± 4.58	31.83 ± 6.27	−44.27 ± 6.45
Rocan 1	CH_1.40_O_0.50_N_0.04_	Duboc et al. (1999), Table 9	−104.58 ± 5.61	28.44 ± 5.60	−67.71 ± 7.28
Rocan BUV 2	CH_1.56_O_0.59_N_0.05_	Duboc et al. (1999), Table 9	−129.66 ± 6.95	32.30 ± 6.36	−87.79 ± 8.85
Scenedesnus obtusiusculus	CH_1.64_O_0.44_N_0.11_	Duboc et al. (1999), Table 9	−98.86 ± 5.30	31.48 ± 6.20	−58.06 ± 7.15
Selenastrum capricornutum	CH_1.60_O_0.43_N_0.08_	Duboc et al. (1999), Table 9	−95.37 ± 5.11	30.26 ± 5.96	−56.14 ± 6.89

**Table 2:** Standard specific (per gram) enthalpy of formation *h*_*f*_*⁰*, entropy *s*⁰, and Gibbs free energy of formation *g*_*f*_*⁰* of dry bacteria, fungi and algae biomass. Empirical formulas reflect elemental composition of microorganisms and are normalized per mole of carbon atoms. To appreciate the size of a cell, a single Magnetospirillum gryphiswaldense cell can be described by C_2.31 × 10_^10^H_4.68 × 10_^10^O_2.93 × 10⁹_N_6.56 × 10_^9^^9^Fe_4.00 × 10⁷_. The thermodynamic parameters for entire cells are given by the equation *X*⁰_*cell*_ = *m*_*bio*_ · *x*⁰_*bio*_ + *m*_*w*_ · *x*_w_⁰ where *X*⁰_*cell*_ is thermodynamic parameter (*H*_*f*_, *S*, or *G*_*f*_) for a single cell, *x*⁰_*bio*_ thermodynamic parameter for biomass from this table, *m*_*bio*_ mass of the cell dry matter, *x*_w_⁰ thermodynamic parameter for water and *m*_*w*_ mass of water in the cell (Section 4.3).

Name	Formula	Reference	*h*_*f*_*⁰* (kJ/g)	*s⁰* (J/g K)	*g*_*f*_*⁰* (kJ/g)
*BACTERIA*					
Bacteria (general)	CH_1.666_O_0.270_N_0.200_	Abbott and Clamen (1973)	−2.97 ± 0.16	1.45 ± 0.29	−1.10 ± 0.24
Aerobacter aerogenes	CH_1.830_O_0.550_N_0.250_	Naresh et al. (2011)	−4.95 ± 0.27	1.47 ± 0.29	−3.04 ± 0.35
Brevibacterium flavum	CH_1.80_O_0.33_N_0.19_	Duboc et al. (1999), Table 9	−3.65 ± 0.20	1.50 ± 0.30	−1.70 ± 0.28
Bacillus cereus	CH_1.49_O_0.43_N_0.22_	Duboc et al. (1999), Table 9	−3.91 ± 0.21	1.34 ± 0.26	−2.18 ± 0.29
Corynebacterium glutamicum	CH_1.78_O_0.44_N_0.24_	Duboc et al. (1999), Table 9	−4.27 ± 0.23	1.47 ± 0.29	−2.37 ± 0.31
Escherichia coli	CH_1.770_O_0.490_N_0.240_	Bauer and Ziv (1976)	−4.57 ± 0.24	1.45 ± 0.29	−2.68 ± 0.33
E. coli	CH_1.74_O_0.34_N_0.22_	Duboc et al. (1999), Table 9	−3.58 ± 0.19	1.47 ± 0.29	−1.68 ± 0.28
E. coli K-12: grown on Acetic acid	CH_1.54_O_0.4_N_0.21_	Battley (1992), Table 3	−3.79 ± 0.20	1.37 ± 0.27	−2.02 ± 0.28
E. coli K-12: grown on glucose	CH_1.74_O_0.464_N_0.26_	Battley (1992), Table 3	−4.32 ± 0.23	1.44 ± 0.28	−2.45 ± 0.32
E. coli K-12: grown on glucose	CH_1.81_O_0.40_N_0.22_	Battley (1992), Table 3	−4.09 ± 0.22	1.49 ± 0.29	−2.16 ± 0.31
E. coli K-12: grown on glucose	CH_1.73_O_0.53_N_0.235_	Battley (1992), Table 3	−4.77 ± 0.26	1.43 ± 0.28	−2.91 ± 0.34
E. coli K-12: grown on glucose	CH_1.78_O_0.511_N_0.237_	Battley (1992), Table 3	−4.71 ± 0.25	1.46 ± 0.29	−2.82 ± 0.34
E. coli K-12: grown on glucose	CH_1.81_O_0.49_N_0.234_	Battley (1992), Table 3	−4.62 ± 0.25	1.47 ± 0.29	−2.72 ± 0.33
E. coli K-12: grown on glucose	CH_1.54_O_0.34_N_0.24_	Battley (1992), Table 3	−3.28 ± 0.18	1.37 ± 0.27	−1.51 ± 0.26
E. coli K-12: grown on Succinic acid	CH_1.56_O_0.36_N_0.23_	Battley (1992), Table 3	−3.48 ± 0.19	1.38 ± 0.27	−1.69 ± 0.27
E. coli W: grown on glucose	CH_1.698_O_0.427_N_0.250_	Battley (1992), Table 3	−4.07 ± 0.22	1.43 ± 0.28	−2.21 ± 0.30
E. coli W: grown on glycerol	CH_1.698_O_0.427_N_0.250_	Battley (1992), Table 3	−4.07 ± 0.22	1.43 ± 0.28	−2.21 ± 0.30
Flavobacterium dehydrogenans	CH_1.63_O_0.40_N_0.21_	Duboc et al. (1999), Table 9	−3.90 ± 0.21	1.41 ± 0.28	−2.07 ± 0.29
Klebsiella aerogenes	CH_1.750_O_0.430_N_0.220_	Naresh et al. (2011)	−4.22 ± 0.23	1.46 ± 0.29	−2.33 ± 0.31
Klebsiella aerogenes	CH_1.730_O_0.430_N_0.240_	Naresh et al. (2011)	−4.15 ± 0.22	1.45 ± 0.29	−2.27 ± 0.31
Klebsiella aerogenes	CH_1.750_O_0.470_N_0.170_	Naresh et al. (2011)	−4.61 ± 0.25	1.46 ± 0.29	−2.72 ± 0.33
Klebsiella aerogenes	CH_1.730_O_0.430_N_0.240_	Naresh et al. (2011)	−4.15 ± 0.22	1.45 ± 0.29	−2.27 ± 0.31
Lactobacillus helveticus	CH_1.58_O_0.39_N_0.23_	Duboc et al. (1999), Table 9	−3.72 ± 0.20	1.38 ± 0.27	−1.93 ± 0.28
Magnetospirillum gryphiswaldense	CH_2.060_O_0.130_N_0.280_Fe_0.00174_	Naresh et al. (2011)	−2.18 ± 0.12	1.67 ± 0.33	−0.02 ± 0.22
Methanobacterium thermoautotrophicum	CH_1.63_O_0.43_N_0.22_	Duboc et al. (1999), Table 9	−4.08 ± 0.22	1.40 ± 0.28	−2.26 ± 0.30
Methylococcus capsulatus	CH_2.000_O_0.500_N_0.270_	van Dijken and Harder (1975)	−4.79 ± 0.26	1.55 ± 0.30	−2.79 ± 0.35
Paracoccus denitrificans	CH_1.510_O_0.460_N_0.190_	Shimizu et al. (1978)	−4.21 ± 0.23	1.35 ± 0.27	−2.47 ± 0.30
Paracoccus denitrificans	CH_1.810_O_0.510_N_0.200_	Stouthamer (1977)	−4.83 ± 0.26	1.47 ± 0.29	−2.92 ± 0.35
Pseudomonas C12B	CH_2.000_O_0.520_N_0.230_	Mayberry et al. (1968)	−5.01 ± 0.27	1.55 ± 0.30	−3.00 ± 0.36
Saccharopolyspora erythraea (a)	CH_1.61_O_0.47_N_0.19_	Duboc et al. (1999), Table 9	−4.39 ± 0.24	1.39 ± 0.27	−2.59 ± 0.32
Saccharopolyspora erythraea (b)	CH_1.68_O_0.47_N_0.16_	Duboc et al. (1999), Table 9	−4.55 ± 0.24	1.43 ± 0.28	−2.70 ± 0.33
*YEAST*					
Saccharomyces cerevisiae	CH_1.613_O_0.557_N_0.158_P_0.012_S_0.003_K_0.022_Mg_0.003_Ca_0.001_	Battley, 1999a,b	−5.04 ± 0.27	1.32 ± 0.26	−3.32 ± 0.35
Saccharomyces cerevisiae	CH_1.640_O_0.520_N_0.160_	Harrison (1967)	−4.82 ± 0.26	1.40 ± 0.28	−3.00 ± 0.34
Saccharomyces cerevisiae	CH_1.830_O_0.560_N_0.170_	Kok and Roels (1980)	−5.22 ± 0.28	1.48 ± 0.29	−3.31 ± 0.37
Saccharomyces cerevisiae	CH_1.810_O_0.510_N_0.170_	Wang et al. (1976)	−4.92 ± 0.26	1.48 ± 0.29	−3.00 ± 0.35
Saccharomyces cerevisiae Whi 2+	CH_1.65_O_0.57_N_0.14_	Duboc et al. (1999), Table 9	−5.17 ± 0.28	1.40 ± 0.28	−3.36 ± 0.36
Saccharomyces cerevisiae Whi 2−	CH_1.64_O_0.5_N_0.18_	Duboc et al. (1999), Table 9	−4.64 ± 0.25	1.40 ± 0.28	−2.82 ± 0.33
Saccharomyces cerevisiae CBS 426a	CH_1.56_O_0.52_N_0.16_	Duboc et al. (1999), Table 9	−4.73 ± 0.25	1.36 ± 0.27	−2.96 ± 0.33
Saccharomyces cerevisiae CBS 426b	CH_1.52_O_0.51_N_0.19_	Duboc et al. (1999), Table 9	−4.54 ± 0.24	1.35 ± 0.27	−2.80 ± 0.32
Candida utilis	CH_1.830_O_0.540_N_0.100_	Herbert (1976)	−5.32 ± 0.29	1.49 ± 0.29	−3.39 ± 0.37
Candida utilis	CH_1.870_O_0.560_N_0.200_	Naresh et al. (2011)	−5.18 ± 0.28	1.49 ± 0.29	−3.25 ± 0.36
Candida utilis	CH_1.830_O_0.460_N_0.190_	Naresh et al. (2011)	−4.58 ± 0.25	1.49 ± 0.29	−2.65 ± 0.33
Candida utilis	CH_1.870_O_0.560_N_0.200_	Naresh et al. (2011)	−5.18 ± 0.28	1.49 ± 0.29	−3.25 ± 0.36
Candida utilis ATCC 9950	CH_1.66_O_0.56_N_0.07_	Duboc et al. (1999), Table 9	−5.34 ± 0.29	1.41 ± 0.28	−3.51 ± 0.37
Candida kefyr NCYC 1441	CH_1.66_O_0.44_N_0.12_	Duboc et al. (1999), Table 9	−4.44 ± 0.24	1.42 ± 0.28	−2.60 ± 0.32
Debaryomyces hansenii	CH_1.71_O_0.6_N_0.10_	Duboc et al. (1999), Table 9	−5.52 ± 0.30	1.42 ± 0.28	−3.68 ± 0.38
Debaryomyces nepaliensis CBS 5921	CH_1.77_O_0.63_N_0.09_	Duboc et al. (1999), Table 9	−5.78 ± 0.31	1.45 ± 0.28	−3.90 ± 0.39
Kluyveromyces marxianus NRRL 665	CH_1.73_O_0.53_N_0.17_	Duboc et al. (1999), Table 9	−4.95 ± 0.27	1.44 ± 0.28	−3.08 ± 0.35
Zygosaccharomyces bailii NCYC 563	CH_1.63_O_0.55_N_0.13_	Duboc et al. (1999), Table 9	−5.07 ± 0.27	1.39 ± 0.27	−3.26 ± 0.35
*FILAMENTOUS FUNGI*					
Aspergillus niger	CH_1.60_O_0.55_N_0.10_	Duboc et al. (1999), Table 9	−5.12 ± 0.27	1.38 ± 0.27	−3.33 ± 0.36
Aspergillus niger (spores)	CH_1.50_O_0.53_N_0.12_	Duboc et al. (1999), Table 9	−4.83 ± 0.26	1.34 ± 0.26	−3.10 ± 0.34
Mucor rouxii	CH_1.79_O_0.43_N_0.07_	Duboc et al. (1999), Table 9	−4.68 ± 0.25	1.49 ± 0.29	−2.74 ± 0.34
Neurospora crassa	CH_1.80_O_0.45_N_0.13_	Duboc et al. (1999), Table 9	−4.65 ± 0.25	1.49 ± 0.29	−2.72 ± 0.34
Penicillium chrysogenum	CH_1.87_O_0.22_N_0.08_	Duboc et al. (1999), Table 9	−3.09 ± 0.17	1.59 ± 0.31	−1.02 ± 0.26
*ALGAE*					
Algae (general)	CH_2.481_O_1.038_N_0.151_P_0.00943_	Wang et al. (2017)	−7.77 ± 0.42	1.61 ± 0.32	−5.68 ± 0.51
Chlamydomonas	CH_1.65_O_0.39_N_0.12_	Duboc et al. (1999), Table 9	−4.08 ± 0.22	1.43 ± 0.28	−2.23 ± 0.30
Chlorella	CH_1.719_O_0.404_N_0.175_P_0.0105_	Manahan and Manahan (2009)	−4.15 ± 0.22	1.44 ± 0.28	−2.29 ± 0.31
Chlorella a sp. MP-1	CH_1.793_O_0.608_N_0.121_	Phukan et al. (2011)	−5.59 ± 0.30	1.46 ± 0.29	−3.70 ± 0.39
Chlorella minutissima	CH_1.714_O_0.286_N_0.143_	Prajapati et al. (2014)	−3.30 ± 0.18	1.48 ± 0.29	−1.38 ± 0.26
Chlorella pyrenoidosa	CH_1.625_O_0.250_N_0.125_	Prajapati et al. (2014)	−2.89 ± 0.16	1.44 ± 0.28	−1.03 ± 0.24
Chlorella vulgaris	CH_1.667_O_0.222_N_0.111_	Prajapati et al. (2014)	−2.73 ± 0.15	1.47 ± 0.29	−0.82 ± 0.23
Chrorella sp. ATCC 7516 (medium 5)	CH_1.76_O_0.35_N_0.09_	Duboc et al. (1999), Table 9	−4.00 ± 0.21	1.49 ± 0.29	−2.07 ± 0.30
Chlorella Spain sp. ATCC 7516 (medium S)	CH_1.78_O_0.36_N_0.12_	Duboc et al. (1999), Table 9	−4.03 ± 0.22	1.50 ± 0.30	−2.08 ± 0.30
Rocan 1	CH_1.40_O_0.50_N_0.04_	Duboc et al. (1999), Table 9	−4.76 ± 0.25	1.29 ± 0.25	−3.08 ± 0.33
Rocan BUV 2	CH_1.56_O_0.59_N_0.05_	Duboc et al. (1999), Table 9	−5.47 ± 0.29	1.36 ± 0.27	−3.70 ± 0.37
Scenedesnus obtusiusculus	CH_1.64_O_0.44_N_0.11_	Duboc et al. (1999), Table 9	−4.44 ± 0.24	1.42 ± 0.28	−2.61 ± 0.32
Selenastrum capricornutum	CH_1.60_O_0.43_N_0.08_	Duboc et al. (1999), Table 9	−4.41 ± 0.24	1.40 ± 0.28	−2.60 ± 0.32

### 2.1 Enthalpy of combustion

Standard molar enthalpy of combustion of microorganism biomass can be calculated from their elemental composition, using empirical relations. The empirical relations predict enthalpy of biomass combustion, from which enthalpy of formation can be estimated using simple thermochemical relations. In the literature there are at least 40 empirical equations for estimating enthalpy of combustion of biomass (Kiang, 2012). This section reviews and compares using experimental data the most widely used methods, including Patel-Erickson, Boie, Dulong, Mason-Gandhi and Channiwala-Parikh equations (Annamalai et al., 2018).

Dulong was the first to propose, in the 19^th^ century, that heat of combustion of coal can be empirically correlated with its chemical composition (Kiang, 2012). Dulong's equation is still in use today and its success has inspired many to look for similar equations applicable to a wide range of fuels, an example being the widely used Boie equation (Annamalai et al., 2018; Kiang, 2012). The underlaying mechanism of the correlation was discovered by Thornton (1917), who realized that the heat of combustion is proportional to number of oxygen atoms used to burn the fuel. Thornton's work was generalized and extended by Patel and Erickson (1981), who realized that enthalpy of combustion – a redox reaction – is proportional to the number of electrons transferred from the fuel to oxygen.

The Patel-Erickson equation states that the standard molar enthalpy of combustion ${\bar{h}}_{C}^{o}$ of organic matter is proportional to the number of electrons that it transfers to oxygen during combustion (Patel and Erickson, 1981). The proportionality is expressed through the equation (Patel and Erickson, 1981; Battley, 1998)

(1) $${\bar{h}}_{C}^{o}=-111.14\;\frac{kJ}{mol}\cdot E$$

where *E* is the number of electrons transferred to oxygen during combustion to CO_2_(g), H_2_O(l), N_2_(g), P_4_O_10_(s) and SO_3_(g) (Patel and Erickson, 1981; Battley, 1998).

There are two conventions about sulphur oxidation: SO_2_ and SO_3_ conventions. The first is used in the combustion literature (van Loo and Koppejan, 2008) and is based on sulphur oxidation to SO_2_

(2) $$\text{S}\;+{\;\text{O}}_{2}\to {\text{SO}}_{2}$$

This convention is due to the fact that in most combustion processes sulphur is oxidized to SO_2_. The second convention is used in the calorimetry literature (Battley, 1992; 1999b; Minas da Piedade, 1999, p. 38) and considers sulphur oxidation to SO_3_

(3) $$\text{S}\;+\;1\text{½}{\;\text{O}}_{2}\to \;{\text{SO}}_{3}$$

The second convention is due to the fact that sulphur is oxidized to SO_3_, H_2_SO_4_ or sulphates in bomb calorimeters (Battley, 1992; 1999b; Minas da Piedade, 1999, p. 38). Boie, Dulong, Mason-Gandhi, and Channiwala-Parikh equations, which will be discussed below, use the S→SO_2_ convention. The Patel-Erickson equation can be used with both conventions. The S→SO_2_ convention implies the coefficient 4 multiplying *n*_*S*_ in Eq. (4), since an S atom gives 4 electrons when it is oxidized to SO_2_. The S→SO_3_ convention requires multiplying *n*_*S*_ by 6, since an S atom gives 6 electrons when it is oxidized to SO_3_. However, regardless of which convention is chosen, the results do not change significantly. The difference between Patel-Erickson equation enthalpies of combustion calculated using the two conventions is 0.13%.

Thus, during combustion, a C atom gives its 4 valence electrons, H gives 1, N gives none since it is converted to N_2_, P gives 5 and S gives 6. Inorganic ions, like Na^+^ and Mg^2+^ are not included, since they are already in their highest oxidation state and cannot transfer any electrons to oxygen (Battley, 1998). Thus, *E* is calculated through the equation

(4) $$E=4\;n_{C}+n_{H}-2\;n_{O}-0\;n_{N}+5\;n_{P}+6\;n_{S}$$

where *n*_*C*_, *n*_*H*_, *n*_*O*_, *n*_*N*_, *n*_*P*_ and *n*_*S*_ are the number of C, H, O, N, P and S atoms in the biomass empirical formula (Patel and Erickson, 1981; Battley, 1998). If any of these atoms are not present, they are just neglected during the calculation (Battley, 1998). An example calculation of biomass ${\bar{h}}_{C}^{o}$ using the Patel-Erickson equation is given in the section 2.5.

The Boie equation is a widely used model for estimating standard specific enthalpy of combustion *h*_*C*_*⁰* of fuels of known elemental composition

(5) $$h_{c}^{o}\left(kJ/kg\right)=-\left(35160\cdot w_{C}+116225\cdot w_{H}-11090\cdot w_{O}+6280\cdot w_{N}+10465\cdot w_{S}\right)$$

where *w*_*C*_, *w*_*H*_, *w*_*O*_, *w*_*N*_ and *w*_*S*_ are mass fractions of carbon, hydrogen, oxygen, nitrogen and sulphur in the fuel, respectively (Annamalai et al., 2018). The method of converting standard enthalpy of combustion from molar ${\bar{h}}_{C}^{o}$ to mass specific *h*_*C*_*⁰* basis is described in section 3.

The Dulong equation gives standard molar enthalpy of combustion *h*_*C*_*⁰* of fuels as a function of their composition as (Annamalai et al., 2018)

(6) $$h_{c}^{o}\left(kJ/kg\right)=-\left(33800\cdot w_{C}+144153\cdot w_{H}-18019\cdot w_{O}+9412\cdot w_{S}\right)$$

The Mason-Gandhi equation gives standard specific enthalpy of combustion as

(7) $$h_{c}^{o}\left(kJ/kg\right)=\{\begin{array}{c} -\left(33610\cdot w_{C}+141830\cdot w_{H}+9420\cdot w_{S}-14510\cdot w_{O}\right)\;\;\;\;\;\;\;\;\;\;\;\;\;\;\;\;\;\;\;\;\;\;\;\;\;\;\;\;\;\;\;\;\;\;\;\;\;\;\;for\;\;w_{O}<0.15\\ -\left[33610\cdot w_{C}+141830\cdot w_{H}+9420\cdot w_{S}-\left(15320-7200\frac{w_{O}}{1-w_{Ash}}\right)\cdot w_{O}\right]\;\;\;\;\;for\;w_{O}>0.15\; \end{array}$$

where *w*_*Ash*_ is the mass fraction of ash in the sample (Annamalai et al., 2018).

The Channiwala-Parikh equation gives standard specific enthalpy of combustion as (Annamalai et al., 2018)

(8) $$h_{c}^{o}\left(kJ/kg\right)=-\left(34910\cdot w_{C}+117830\cdot w_{H}-10340\cdot w_{O}-2110\cdot w_{Ash}+10050\cdot w_{S}-1510\cdot w_{N}\right)$$

The five models were compared to experimental heat of combustion data, reported by Duboc et al. (1999) and Battley (1999b). Microorganism compositions in the form of mass fractions and empirical formulas (UCF) were taken from Duboc et al. (1999) and Battley (1999b). They are given in Table 3. The mass fraction data was used to calculate enthalpies of combustion of the microorganisms, using the Boie, Dulong, Mason-Gandhi, and Channiwala-Parikh equations. The empirical formulas were used to calculate the enthalpies of combustion using the Patel-Erickson equation. The results are presented in Table 4. Based on the results from Table 4, average absolute deviations were calculated and are presented in Table 5. As can be seen from Table 5, the best results were obtained from the Channiwala-Parikh equation and the Patel-Erickson equation. This result is in agreement with that of Cordier et al. (1987), who found the Patel-Erickson equation to be among the most precise models for estimating enthalpy of combustion of microbial biomass.

**Table 3:** Elemental composition of dry microorganism biomass: element mass fractions, elemental empirical formulas and empirical formula molar masses. Data taken from taken from Duboc *et al.* (1999) and Battley (1999b). *w*_*Ash*_, *w*_*C*_, *w*_*H*_, *w*_*O*_ and *w*_*N*_ represent mass fractions of ash, C, H, O and N, respectively, while *Mr* is the empirical formula molar mass, which can be calculated through the equation: *Mr* = 12.0107 / *w_C_* (Duboc *et al.* (1999)).

Name	w_Ash_	w_c_	w_H_	w_O_	w_N_	Empirical formula	Mr (g/mol)
*Bacteria*							
Lactobacillus helveticus	0.0903	0.4754	0.0625	0.2439	0.1279	CH_1.58_O_0.39_N_0.23_	25.26
F. dehydrogenans	0.1353	0.4516	0.0615	0.2429	0.1087	CH_1.63_O_0.40_N_0.21_	26.60
Saccharopolyspora erythraea (a)	0.0932	0.4586	0.0616	0.2861	0.1005	CH_1.61_O_0.47_N_0.19_	26.19
Saccharopolyspora erythraea (b)	0.0483	0.4848	0.068	0.3067	0.0922	CH_1.68_O_0.47_N_0.16_	24.77
B. flavum	0.3023	0.3854	0.0578	0.1698	0.0847	CH_1.80_O_0.33_N_0.19_	31.16
Escherichia coli	0.1127	0.4783	0.0695	0.2165	0.123	CH_1.74_O_0.34_N_0.22_	25.11
Bacillus cereus	0.0998	0.4605	0.0573	0.2626	0.1198	CH_1.49_O_0.43_N_0.22_	26.08
Corynebacterium glutamicum	0.3209	0.3365	0.05	0.1993	0.0933	CH_1.78_O_0.44_N_0.24_	35.69
Methanobacterium thermoautotrophicum	0.1865	0.412	0.0558	0.239	0.1067	CH_1.63_O_0.43_N_0.22_	29.15
*Algae*							
Rocan 1	0.0615	0.5118	0.0596	0.3405	0.0266	CH_1.40_O_0.50_N_0.04_	23.47
Rocan BUV 2	0.1167	0.4452	0.0578	0.3519	0.0284	CH_1.56_O_0.59_N_0.05_	26.98
Chlamydomonas	0.042	0.5326	0.0734	0.2795	0.0725	CH_1.65_O_0.39_N_0.12_	22.55
Chrorella sp. ATCC 7516 (medium 5)	0.0543	0.5505	0.0806	0.2599	0.0547	CH_1.76_O_0.35_N_0.09_	21.82
Chlorella Spain sp. ATCC 7516 (medium S)	0.0431	0.539	0.08	0.2604	0.0775	CH_1.78_O_0.36_N_0.12_	22.28
Selenastrum capricornutum	0.0565	0.5238	0.07	0.3028	0.0469	CH_1.60_O_0.43_N_0.08_	22.93
Scenedesnus obtusiusculus	0.0522	0.5104	0.0697	0.3011	0.0666	CH_1.64_O_0.44_N_0.11_	23.53
*Filamentous fungi*							
Neurospora crassa	0.0848	0.4817	0.0722	0.2899	0.0714	CH_1.80_O_0.45_N_0.13_	24.93
Penicillium chrysogenum	0.2113	0.5114	0.0795	0.15	0.0478	CH_1.87_O_0.22_N_0.08_	23.49
Mucor rouxii	0.0957	0.5035	0.0752	0.2865	0.0391	CH_1.79_O_0.43_N_0.07_	23.85
Aspergillus niger	0.0877	0.4618	0.0617	0.3372	0.0516	CH_1.60_O_0.55_N_0.10_	26.01
Aspergillus niger (spores)	0.0318	0.4915	0.0614	0.3483	0.067	CH_1.50_O_0.53_N_0.12_	24.44
*Yeast*							
Candida kefyr NCYC 1441	0.047	0.5118	0.0713	0.2992	0.0707	CH_1.66_O_0.44_N_0.12_	23.47
Candida utilis ATCC 9950	0.0968	0.4597	0.0639	0.342	0.0376	CH_1.66_O_0.56_N_0.07_	26.13
Debaryomyces hansenii	0.0874	0.4441	0.0636	0.3522	0.0527	CH_1.71_O_0.6_N_0.10_	27.05
D. nepaliensis CBS 5921	0.0451	0.4567	0.0678	0.383	0.0474	CH_1.77_O_0.63_N_0.09_	26.30
Saccharomyces cerevisae	0.0502	0.4588	0.0621	0.3403	0.0847	CH_1.613_O_0.557_N_0.158_P_0.012_S_0.003_K_0.022_Mg_0.003_Ca_0.001_	26.18
S. cerevisiae Whi 2+	0.0704	0.4516	0.0627	0.3412	0.0741	CH_1.65_O_0.57_N_0.14_	26.60
S. cerevisiae Whi 2-	0.0864	0.4544	0.0624	0.3022	0.0946	CH_1.64_O_0.5_N_0.18_	26.43
S. cerevisiae CBS 426a	0.0955	0.4505	0.0589	0.31	0.0851	CH_1.56_O_0.52_N_0.16_	26.66
S. cerevisiae CBS 426b	0.0306	0.4777	0.0611	0.3268	0.1038	CH_1.52_O_0.51_N_0.19_	25.14
Kluyveromyces marxianus NRRL 665	0.1005	0.4385	0.0635	0.3107	0.0868	CH_1.73_O_0.53_N_0.17_	27.39
Zygosaccharomyces bailii NCYC 563	0.065	0.4626	0.0632	0.3372	0.072	CH_1.63_O_0.55_N_0.13_	25.96

**Table 4:** Standard specific enthalpy of combustion of dry microorganism biomass. The experimental values were taken from Duboc et al. (1999) and Battley (1999b). The theoretical values were calculated using the Patel-Erickson, Boie, Dulong, Mason-Gandhi and Channiwala-Parikh equations.

Name	h_C_⁰ (kJ/kg)					
	Experiment	Patel-Erickson	Boie	Dulong	Mason-Gandhi	Channiwala-Parikh
Lactobacillus helveticus	−21274.9881355791	−21115.55	−22077.49	−20683.25	−21576.84	−21055.00
F. dehydrogenans	−19540.6196141774	−20183.81	−21014.97	−19752.67	−20670.87	−20050.70
Saccharopolyspora erythraea (a)	−19824.416561899	−19817.69	−20742.13	−19225.27	−20417.14	−19961.37
Saccharopolyspora erythraea (b)	−21279.9054176692	−21263.91	−22126.58	−20662.22	−21951.56	−21524.40
B. flavum	−15777.6965539061	−18330.61	−18917.30	−18298.94	−18847.27	−17743.41
Escherichia coli	−21106.0970634518	−22395.10	−23266.12	−22284.06	−22996.41	−22224.50
Corynebacterium glutamicum	−19469.4647272848	−19729.35	−20690.98	−19093.08	−20132.78	−19720.95
Methanobacterium thermoautotrophicum	−15061.7699218197	−15257.49	−16018.28	−14990.16	−15769.12	−14759.97
Rocan 1	−16235.4900213976	−18185.18	−18990.84	−17662.80	−18605.51	−17931.94
Rocan BUV 2	−20232.1796398212	−20837.95	−21312.80	−19754.89	−21327.68	−21198.91
Chlamydomonas	−19845.6443004987	−18043.94	−18646.82	−17038.92	−18779.24	−18424.74
Chrorella sp. ATCC 7516 (medium 5)	−24553.1584337299	−24001.16	−24612.78	−23546.40	−24616.19	−24153.66
Chlorella Spain sp. ATCC 7516 (medium S)	−26735.0487481995	−25775.67	−26184.54	−25542.49	−26466.41	−25830.52
Selenastrum capricornutum	−25323.8945273797	−25237.21	−25848.10	−25058.29	−25983.07	−25342.39
Scenedesnus obtusiusculus	−22935.0845496099	−22974.49	−23489.04	−22339.00	−23593.81	−23212.97
Neurospora crassa	−24791.8406087905	−22481.21	−23125.60	−21873.46	−23115.95	−22706.73
Penicillium chrysogenum	−25543.4512559634	−21841.11	−22561.42	−21465.60	−22649.96	−22039.17
Mucor rouxii	−22890.3094740523	−25695.83	−25857.40	−26042.63	−26371.04	−25151.44
Aspergillus niger	−20897.595477366	−22969.34	−23511.44	−22696.16	−23852.61	−23214.62
Aspergillus niger (spores)	−16098.6170664491	−19229.52	−19992.47	−18427.07	−20003.47	−19641.94
Candida kefyr NCYC 1441	−21565.8121508322	−20193.36	−20975.47	−19187.68	−20793.86	−20623.34
Candida utilis ATCC 9950	−21902.570208231	−22637.59	−23407.60	−22185.66	−23406.67	−22968.56
Debaryomyces hansenii	−20591.5225590515	−19312.23	−20033.18	−18586.74	−20206.41	−19780.16
D. nepaliensis CBS 5921	−19412.0659078988	−18533.58	−19431.52	−17832.42	−19529.54	−19091.78
Saccharomyces cerevisae	−18784.067539777	−19391.25	−20323.05	−18670.34	−20221.32	−19863.70
S. cerevisiae Whi 2+	−19288.7009083567	−19059.42	−19987.83	−18308.76	−20204.24	−19805.32
S. cerevisiae Whi 2−	−19597.4589324519	−18846.58	−19847.00	−18154.39	−19745.52	−19364.85
S. cerevisiae CBS 426a	−19841.8493509954	−19510.05	−20471.83	−18908.53	−20212.59	−19765.80
S. cerevisiae CBS 426b	−20085.2989417769	−18842.36	−19781.76	−18131.62	−19510.87	−19131.74
Kluyveromyces marxianus NRRL 665	−19970.484651186	−19891.61	−20924.93	−19065.40	−20507.95	−20275.50
Zygosaccharomyces bailii NCYC 563	−19440	−18949.10	−19897.39	−18376.51	−19756.97	−19234.48

**Table 5:** A comparison of five predictive enthalpy of combustion models, based on absolute average deviations from experimental data.

Equation	AAD (%)
Channiwala-Parikh	5.00
Patel-Erickson	5.36
Mason-Ghandi	6.15
Boie	6.56
Dulong	6.65

The Patel-Erickson equation was chosen as the method to calculate enthalpies of combustion of microorganisms presented in this study for three reasons:

1. It is among the most precise equations for estimating microorganism enthalpy of combustion.
2. It is the most widely applicable equation for animate matter, since it includes phosphorus, which is present in all living organisms. Phosphorus in living organisms is oxidized during combustion to P_4_O_10_, which has a very negative enthalpy of formation (*(*${\bar{h}}_{f}^{o}$*)*_*P4O10*_ = −2984.0 kJ/mol, compared with *(*${\bar{h}}_{f}^{o}$*)*_*CO2*_ = −393.51 kJ/mol (Atkins and de Paula, 2006). Thus, phosphorus has a great influence on calculated enthalpy of formation of microorganisms, on which it affects through Eq. (10).
3. It can be used with any kind of elemental analysis, even those that do not determine ash content of a sample. Such methods are often used in biomedical studies, an example being *in vivo neutron activation analysis*, a noninvasive method that allows determination of elemental compositions of living organisms (Heymsfield et al., 1993).

Thus, based on the Patel-Erickson equation, enthalpies of combustion of microorganisms were calculated, and then converted into enthalpies of formation.

### 2.2 Enthalpy of formation

Once ${\bar{h}}_{C}^{o}$ is determined, standard molar enthalpy of formation of microorganism biomass *(*${\bar{h}}_{f}^{o}$*)*_*bio*_ can be calculated as enthalpy of the first reactant in the oxidation reaction (Battley, 1998)

(9) C_nC_H_nH_O_nO_N_nN_P_nP_S_nS_K_nK_Mg_nMg_Ca_nCa_Fe_nFe_ + (n_C_ + ¼ n_H_ + 1¼ n_P_ + 1½ n_S_ + ¼ n_K_ + ½ n_Mg_ + ½ n_Ca_ + ¾ n_Fe_ - ½ n_O_) O_2_ → n_C_ CO_2_ + ½ n_H_ H_2_O + ½ n_N_ N_2_ + ¼ n_P_ P_4_O_10_ + n_S_ SO_3_ + ½ n_K_ K_2_O + n_Mg_ MgO + n_Ca_ CaO + ½ n_Fe_ Fe_2_O_3_

that is, using the formula

(10) $${\left({\bar{h}}_{f}^{o}\right)}_{bio}=n_{C}{\left({\bar{h}}_{f}^{o}\right)}_{CO2}+\frac{1}{2}n_{H}{\left({\bar{h}}_{f}^{o}\right)}_{H2O}+\frac{1}{4}n_{P}{\left({\bar{h}}_{f}^{o}\right)}_{P4O10}+n_{S}{\left({\bar{h}}_{f}^{o}\right)}_{SO3}+\frac{1}{2}n_{K}{\left({\bar{h}}_{f}^{o}\right)}_{K2O}+n_{Mg}{\left({\bar{h}}_{f}^{o}\right)}_{MgO}+n_{Ca}{\left({\bar{h}}_{f}^{o}\right)}_{CaO}+\frac{1}{2}n_{Fe}{\left({\bar{h}}_{f}^{o}\right)}_{Fe2O3}-{\bar{h}}_{C}^{o}$$

More details on how to calculate biomass enthalpy of formation in practice and an example calculation of *(*${\bar{h}}_{f}^{o}$*)*_*bio*_ for *E. coli* can be found in section 2.5.

As was shown above, the error in enthalpy of combustion estimated through Patel's rule is 5.36% *(*${\bar{h}}_{f}^{o}$*)*_*bio*_ is calculated using ${\bar{h}}_{c}^{o}$ and ${\bar{h}}_{f}^{o}$ of the oxides. The oxide ${\bar{h}}_{f}^{o}$ values have been accurately determined by experiment and have a negligible compared to the error in ${\bar{h}}_{c}^{o}$. Thus, the absolute error in *(*${\bar{h}}_{f}^{o}$*)*_*bio*_ is equal to that of ${\bar{h}}_{c}^{o}$ estimated from the Patel-Erickson equation.

### 2.3 Entropy

Composition of dry biomass can be used to calculate its standard molar entropy ${\bar{s}}_{bio}^{o}$, through the Battley equation (Battley, 1999a)

(11) $${\bar{s}}_{bio}^{o}=0.187\underset{i}{\sum }\frac{{\bar{s}}_{i}^{o}}{a_{i}}n_{i}$$

where *n*_*i*_ is the number of atoms of element i in the empirical formula of the biomass, ${\bar{s}}_{i}^{o}$ is standard molar entropy of element i and *a*_*i*_ is the number of atoms per molecule of element i in its standard state elemental form. For example, the standard state elemental form of carbon is graphite, which is simply written as C, which makes *a*_*C*_ = 1. On the other hand, hydrogen, oxygen and nitrogen are in their standard state elemental forms all diatomic gasses H_2_, O_2_ and N_2_, respectively, which implies that *a*_*H*_ = *a*_*O*_ = *a*_*N*_ = 2. The summation is over all elements constituting the dry biomass. More details on how to practically apply the Battley equation and an example calculation of ${\bar{s}}_{bio}^{o}$ for *E. coli* can be found in section 2.5.

The Battley equation simply states that standard molar entropy of biomass equals a constant 0.187 times the standard molar entropy of its constituent elements – the sum term. In the sum term, ${\bar{s}}_{i}^{o}$/*a*_*i*_ represents entropy per mole of atoms of an element. The entropy per mole of atoms of the element is then multiplied by the number of moles of that element in the biomass *n*_*i*_, giving the contribution of that element to the entropy of the biomass. Finally, when contributions of all elements are summed, they are multiplied by the constant 0.187, which takes into account the fact that the elements are no longer in their standard state pure forms, but are a part of the biomass. The Battley equation is a consequence of additivity of entropy: entropy of biomass is a sum of contributions of all its constituent elements.

The Battley equation can be used to predict standard specific entropy of a wide range of organic substances. It has been shown to be applicable to dry microorganism biomass, proteins, amino acids, nucleotides and fatty acids (Battley, 1999a). The error of predicting entropy of dry biomass using the Battley equation is 2% (Battley, 1999a). In case of hydrated biomass, the entropy of hydration is the greatest source of error and increases it to 19.7% (Battley, 1999a).

The Battley equation can also be used to find standard molar entropy *of formation* ${\left({\bar{s}}_{f}^{o}\right)}_{bio}$ of dry biomass from elements (Battley, 1999a). In this case, it takes the form (Battley, 1999a)

(12) $${\left({\bar{s}}_{f}^{o}\right)}_{bio}=-0.813\underset{i}{\sum }\frac{{\bar{s}}_{i}^{o}}{a_{i}}n_{i}$$

Eq. (12) is based on the definition of entropy of formation and the Battley equation. The formation of biomass from elements can be, in a very simplified way, represented by the chemical equation

(13) (elements)→(biomass)

Thus the entropy of formation of biomass is ${\left({\bar{s}}_{f}^{o}\right)}_{bio}={\bar{s}}_{bio}^{o}-{\bar{s}}_{elements}^{o}$ = ${\bar{s}}_{bio}^{o}-\bar{s}elementso$. As was explained above, ${\bar{s}}_{elements}^{o}$ is the sum term in the Battley equation: ${\bar{s}}_{elements}^{o}$ = Σ_i_ (${\bar{s}}_{i}^{o}$/*a*_*i*_)·*n*_*i*_ (Battley, 1999a). The entropy of biomass is ${\bar{s}}_{bio}^{o}$ = 0.187 ${\bar{s}}_{elements}^{o}$. Taking the difference between ${\bar{s}}_{bio}^{o}$ and ${\bar{s}}_{elements}^{o}$ results in the ${\left({\bar{s}}_{f}^{o}\right)}_{bio}$ Eq. (12) (Battley, 1999a).

Since ${\bar{s}}_{elements}^{o}$ is based on experimentally highly accurately determined element entropies, the error in ${\left({\bar{s}}_{f}^{o}\right)}_{bio}$ originates from error in ${\bar{s}}_{bio}^{o}$. Thus the error in ${\left({\bar{s}}_{f}^{o}\right)}_{bio}$ is equal to the error in ${\bar{s}}_{bio}^{o}$ given by the Battley equation.

### 2.4 Gibbs free energy

From *(*${\bar{h}}_{f}^{o}$*)*_*bio*_ and ${\left({\bar{s}}_{f}^{o}\right)}_{bio}$, it is possible to calculate the standard molar Gibbs free energy of formation of microorganisms from elements ${\left({\bar{g}}_{f}^{o}\right)}_{bio}$, using the Gibbs equation

(14) $${\left({\bar{g}}_{f}^{o}\right)}_{bio}={\left({\bar{h}}_{f}^{o}\right)}_{bio}-T\;{\left({\bar{s}}_{f}^{o}\right)}_{bio}$$

where under standard conditions *T* = 298.15 K. The error in ${\left({\bar{g}}_{f}^{o}\right)}_{bio}$, σ_*G*_ can be estimated from error in *(*${\bar{h}}_{f}^{o}$*)*_*bio*_, σ_*H*_, and ${\left({\bar{s}}_{f}^{o}\right)}_{bio}$, σ_*S*_, using the equation σ_*G*_ = σ_*H*_ + *T* σ_*S*_.

### 2.5 An example of estimation of thermodynamic properties of microorganisms

This section gives a practical example, on dry *E. coli* biomass, of estimation of standard specific enthalpy of combustion, enthalpy of formation and entropy.

#### 2.5.1 Standard specific enthalpy of combustion and enthalpy of formation

Enthalpy of formation can be estimated from elemental composition of dry microorganisms using the Patel-Erickson equation. First, elemental composition will be used to find enthalpy of combustion through the Patel-Erickson equation. Then enthalpy of combustion will be used to find enthalpy of formation of dry *E. coli* biomass through classical reaction thermochemistry.

The Patel-Erickson equation is first used to determine the standard specific enthalpy of combustion ${\bar{h}}_{C}^{o}$ for dry *E. coli* biomass. From Table 1, the empirical formula of dry *E. coli* biomass is CH_1.770_O_0.490_N_0.240_. It contains *n*_*C*_ = 1 carbon atoms, *n*_*H*_ = 1.770 hydrogen atoms, *n*_*O*_ = 0.490 oxygen atoms and *n*_*N*_ = 0.240 nitrogen atoms. The number of electrons transferred to oxygen during combustion, *E*, can be determined through Eq. (4). Substituting the actual amounts of C, H, O and N gives

(15) E=4⋅1+1.770−2⋅0.490−0⋅0.240+5⋅0+6⋅0

(16) E=4.790

This result is now substituted into the Patel-Erickson equation (Equation 1) to find ${\bar{h}}_{C}^{o}$ for dry *E. coli* biomass

(17) $${\bar{h}}_{C}^{o}=-111.14\frac{kJ}{mol}\;\;\cdot E$$

(18) $${\bar{h}}_{C}^{o}=-111.14\frac{kJ}{mol}\;\;\cdot 4.790$$

(19) $${\bar{h}}_{C}^{o}=-532.36\frac{kJ}{mol}\;\;$$

Now that ${\bar{h}}_{C}^{o}$ has been determined, the second step is to find the standard specific enthalpy *h*_*f*_*⁰* of dry *E. coli* biomass. The complete combustion of the dry biomass can be represented by reaction (9). Having in mind that *h*_*f*_*⁰* for N_2_ and O_2_ is zero, the enthalpy change for this reaction is ${\bar{h}}_{C}^{o}$ and is given by the equation

(20) $${\bar{h}}_{C}^{o}=n_{C}{\left({\bar{h}}_{f}^{o}\right)}_{CO2}+\frac{1}{2}n_{H}{\left({\bar{h}}_{f}^{o}\right)}_{H2O}+\frac{1}{4}n_{P}{\left({\bar{h}}_{f}^{o}\right)}_{P4O10}+n_{S}{\left({\bar{h}}_{f}^{o}\right)}_{SO3}+\frac{1}{2}n_{K}{\left({\bar{h}}_{f}^{o}\right)}_{K2O}+n_{Mg}{\left({\bar{h}}_{f}^{o}\right)}_{MgO}+n_{Ca}{\left({\bar{h}}_{f}^{o}\right)}_{CaO}+\frac{1}{2}n_{Fe}{\left({\bar{h}}_{f}^{o}\right)}_{Fe2O3}-{\left({\bar{h}}_{f}^{o}\right)}_{bio}$$

which can be manipulated to give the enthalpy of microorganism formation (${\bar{h}}_{f}^{o}$)_bio_

(21) $${\left({\bar{h}}_{f}^{o}\right)}_{bio}=n_{C}{\left({\bar{h}}_{f}^{o}\right)}_{CO2}+\frac{1}{2}n_{H}{\left({\bar{h}}_{f}^{o}\right)}_{H2O}+\frac{1}{4}n_{P}{\left({\bar{h}}_{f}^{o}\right)}_{P4O10}+n_{S}{\left({\bar{h}}_{f}^{o}\right)}_{SO3}+\frac{1}{2}n_{K}{\left({\bar{h}}_{f}^{o}\right)}_{K2O}+n_{Mg}{\left({\bar{h}}_{f}^{o}\right)}_{MgO}+n_{Ca}{\left({\bar{h}}_{f}^{o}\right)}_{CaO}+\frac{1}{2}n_{Fe}{\left({\bar{h}}_{f}^{o}\right)}_{Fe2O3}-{\bar{h}}_{C}^{o}$$

For dry *E. coli* biomass Eqs (9) and (21) become

(22) $${\text{CH}}_{1.770}{\text{O}}_{0.490}{\text{N}}_{0.240}\;+\;2.395{\;\text{O}}_{2}\;\to \;{\text{CO}}_{2}\;+\;0.885{\;\text{H}}_{2}\text{O}\;+\;0.12{\;\text{N}}_{2}$$

(23) $${\left({\bar{h}}_{f}^{o}\right)}_{bio}=1\cdot {\left({\bar{h}}_{f}^{o}\right)}_{CO2}+0.885\cdot {\left({\bar{h}}_{f}^{o}\right)}_{H2O}-{\bar{h}}_{C}^{o}$$

The enthalpies of formation of the inorganic compounds are (${\bar{h}}_{f}^{o}$)_CO2_ = −393.51 kJ/mol and (${\bar{h}}_{f}^{o}$)_H2O_ = −285.83 kJ/mol (Atkins and de Paula, 2006). Substituting these values into Eq. (23), along with the previously calculated ${\bar{h}}_{C}^{o}$ = −532.36 kJ/mol gives

(24) $${\left({\bar{h}}_{f}^{o}\right)}_{bio}=1\cdot \left(-393.51\frac{\text{kJ}}{\text{mol}}\right)+0.885\cdot \left(-285.83\frac{\text{kJ}}{\text{mol}}\right)-\left(-532.36\;\frac{\text{kJ}}{\text{mol}}\right)$$

(25) $${\left({\bar{h}}_{f}^{o}\right)}_{bio}=-114.11\;\frac{\text{kJ}}{\text{mol}}$$

Thus the standard specific enthalpy of formation of dry E. coli biomass is (${\bar{h}}_{f}^{o}$)_*bio*_ = −114.11 kJ/mol. Standard specific enthalpy of formation per unit mass (*h*_*f*_*⁰*)_*bio*_ can be found through the equation

(26) $${\left(h_{f}^{o}\right)}_{bio}=\frac{{\left({\bar{h}}_{f}^{o}\right)}_{bio}}{{\left(M_{r}\right)}_{bio}}$$

where (*M*_*r*_)_*bio*_ is the molar mass of the E. coli empirical formula CH_1.770_O_0.490_N_0.240_: (*M*_*r*_)_*bio*_ = 25.00 g/mol. Thus,

(27) $${\left(h_{f}^{o}\right)}_{bio}=\frac{-114.11\;\frac{\text{kJ}}{\text{mol}}}{25.00\frac{\text{g}}{\text{mol}}}$$

(28) $${\left(h_{f}^{o}\right)}_{bio}=-4.57\;\frac{\text{kJ}}{g}$$

Thus, the standard specific enthalpy of formation of dry E. coli biomass is (*h*_*f*_*⁰*)_*bio*_ = −4.57 kJ/g.

#### 2.5.2 Standard specific entropy

Biomass composition can be used to calculate its standard molar entropy ${\bar{s}}_{bio}^{o}$ through the Battley equation (equation 11). A general microorganism empirical formula is C_nC_H_nH_O_nO_N_nN_P_nP_S_nS_K_nK_Mg_nMg_Ca_nCa_Fe_nFe_. In their standard elemental states C, P, S, K, Mg, Ca and Fe are all solids and are represented by a single atom unit formula (e.g. C_(graphite)_ or K_(s)_) (Atkins and de Paula, 2006). Thus, *a*_*C*_ = *a*_*P*_ = *a*_*S*_ = *a*_*K*_ = *a*_*Mg*_ = *a*_*Ca*_ = *a*_*Fe*_ = 1. On the other hand, H, O and N come in the form of diatomic molecules H_2_, O_2_ and N_2_, thus *a*_*H*_ = *a*_*O*_ = *a*_*N*_ = 2. The Battley equation now becomes

(29) $${\bar{s}}_{bio}^{o}=0.187\left({\bar{s}}_{C}^{o}n_{C}+\frac{{\bar{s}}_{H2}^{o}}{2}n_{H}+\frac{{\bar{s}}_{O2}^{o}}{2}n_{O}+\frac{{\bar{s}}_{N2}^{o}}{2}n_{N}+{\bar{s}}_{P}^{o}n_{P}+{\bar{s}}_{S}^{o}n_{S}+{\bar{s}}_{K}^{o}n_{K}+{\bar{s}}_{Mg}^{o}n_{Mg}+{\bar{s}}_{Ca}^{o}n_{Ca}+{\bar{s}}_{Fe}^{o}n_{Fe}\right)$$

The *E.* coli biomass has the empirical formula is CH_1.770_O_0.490_N_0.240_. Since there are only 4 elements in the empirical formula, Eq. (29) becomes

(30) $${\bar{s}}_{bio}^{o}=0.187\left(n_{C}{\bar{s}}_{C}^{o}+n_{H}\frac{{\bar{s}}_{H2}^{o}}{2}+n_{O}\frac{{\bar{s}}_{O2}^{o}}{2}+n_{N}\frac{{\bar{s}}_{N2}^{o}}{2}\right)$$

Since the empirical formula contains *n*_*C*_ = 1 carbon atoms, *n*_*H*_ = 1.770 hydrogen atoms, *n*_*O*_ = 0.490 oxygen atoms and *n*_*N*_ = 0.240 nitrogen atoms

(31) $${\bar{s}}_{bio}^{o}=0.187\left(1\cdot {\bar{s}}_{C}^{o}+1.770\cdot \frac{{\bar{s}}_{H2}^{o}}{2}+0.490\cdot \frac{{\bar{s}}_{O2}^{o}}{2}+0.240\cdot \frac{{\bar{s}}_{N2}^{o}}{2}\right)$$

The standard molar entropies are ${\bar{s}}_{c}^{o}$ = 5.51 J/mol K, ${\bar{s}}_{\text{H}2}^{o}$ = 130.68 J/mol K, ${\bar{s}}_{\text{O}2}^{o}$ = 205.15 J/mol K and ${\bar{s}}_{\text{N}2}^{o}$ = 191.61 J/mol K (Atkins and de Paula, 2006). Thus,

(32) $${\bar{s}}_{bio}^{o}=0.187\left(1\cdot 5.51\frac{J}{mol\;K}+1.770\cdot \frac{130.68\frac{J}{mol\;K}}{2}+0.490\cdot \frac{205.15\frac{J}{mol\;K}}{2}+0.240\cdot \frac{191.61\frac{J}{mol\;K}}{2}\right)$$

(33) $${\bar{s}}_{bio}^{o}=0.187\cdot 194.42\frac{J}{mol\;K}$$

(34) $${\bar{s}}_{bio}^{o}=36.36\frac{J}{mol\;K}$$

Thus, the standard molar entropy of dry *E. coli* biomass is 36.36 J/mol K. The standard specific entropy *s⁰*_*bio*_ can be found through the equation

(35) $$s_{bio}^{o}=\frac{{\bar{s}}_{bio}^{o}}{{\left(M_{r}\right)}_{bio}}$$

where (*M*_*r*_)_*bio*_ is the molar mass of the *E. coli* empirical formula: (*M*_*r*_)_*bio*_ = 25.00 g/mol. Thus

(36) $$s_{bio}^{o}=\frac{36.36\frac{J}{mol\;K}}{25.00\frac{\text{g}}{\text{mol}}}$$

(37) $$s_{bio}^{o}=1.45\frac{J}{g\;K}$$

Thus, the standard specific entropy of dry *E. coli* cells is 1.45 J/g K.

## 3 Results

Standard molar thermodynamic parameters of dry microorganism biomass have been calculated, as described in section 2, including enthalpy of formation from elements *(*${\bar{h}}_{f}^{o}$*)*_*bio*_, entropy ${\bar{s}}_{bio}^{o}$ and Gibbs free energy of formation from elements ${\left({\bar{g}}_{f}^{o}\right)}_{bio}$. They are given in Table 1. From the standard molar thermodynamic properties, it is possible to calculate standard specific (per gram) thermodynamic properties using the equation *x*⁰ = ${\bar{x}}^{o}$/*M*_*r*_, where *x*⁰ is standard property (*h*_*f*_, *s*, or *g*_*f*_) per gram, $x^{o}$ is the corresponding property per mole and *M*_*r*_ is molar mass of the biomass empirical formula. Thus, standard specific enthalpy of formation from elements *(h*_*f*_*⁰)*_*bio*_, specific entropy *s*⁰_*bio*_ and specific Gibbs free energy of formation from elements *(g*_*f*_*⁰)*_*bio*_ of dry biomass were calculated and are given in Table 2.

**Fig. 1:**
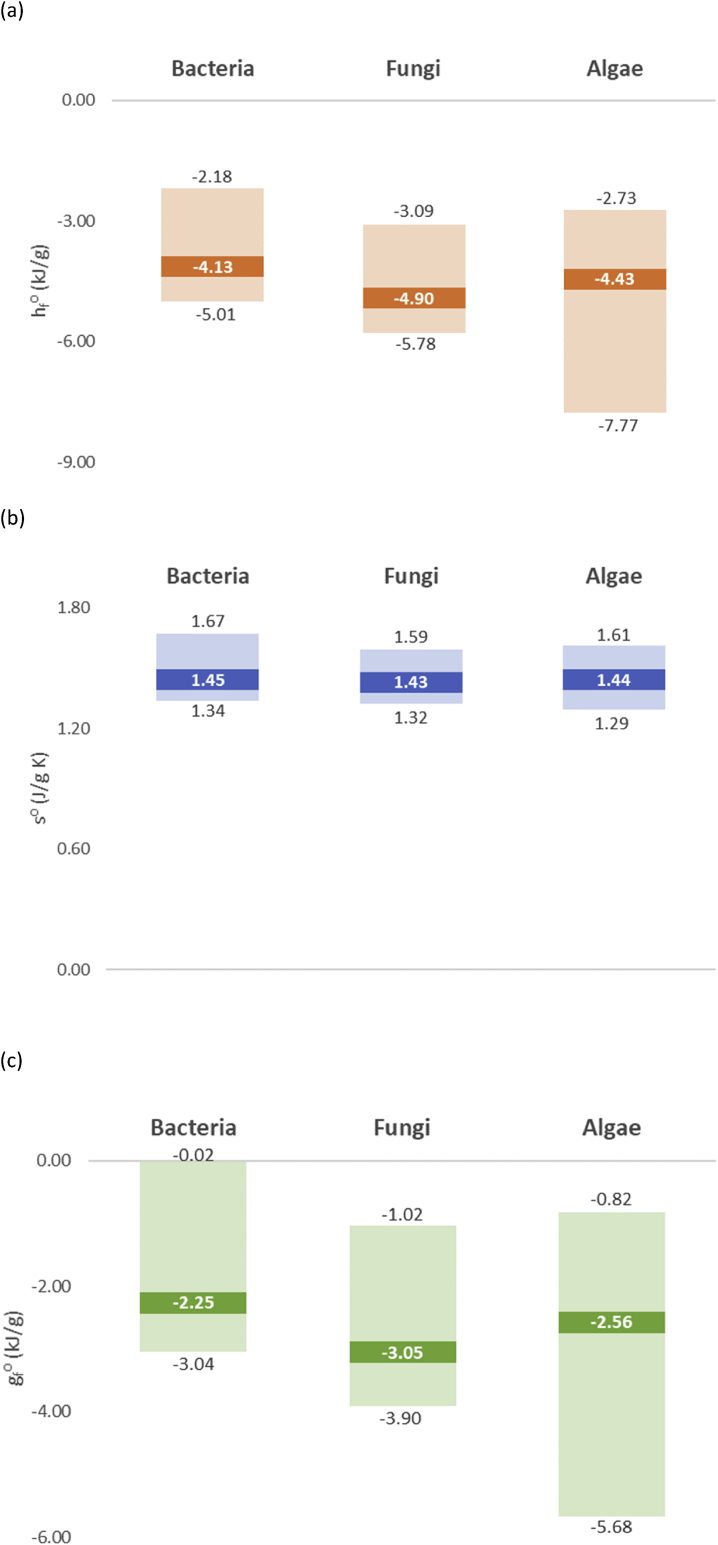
A comparison between average (a) enthalpy of formation from elements, (b) entropy and (c) Gibbs free energy of formation of bacteria, fungi and algae. The top number represents maximal value, middle number is the average value, while the bottom number is the minimal value for the group.

The thermodynamic parameters have very similar values for the three classes, as would be expected. They are shown in Fig. 1. Interesting trends can be seen among the standard thermodynamic properties. For all the studied microorganisms *(h*_*f*_*⁰)*_*bio*_ < 0, *s*⁰_*bio*_ > 0 and *(g*_*f*_*⁰)*_*bio*_ < 0. The *(h*_*f*_*⁰)*_*bio*_ < 0 trend means that formation of biomass from elements is exothermic. The *s*⁰_*bio*_ > 0 trend is a consequence of the third law of thermodynamics, stating that entropy cannot have a negative value. Finally, the *(g*_*f*_*⁰)*_*bio*_ < 0 implies that formation of biomass from elements is a spontaneous process for all the studied microorganism species.

The thermodynamic properties in Tables 1 and 2 are at standard conditions, implying a temperature of 25 °C (298.15 K). To find their values at 37 °C (310.15 K), a correction must be made. Thus, specific enthalpy of formation from elements at 37 °C, *h*_*f*_^*37C*^*,* and specific entropy at 37 °C, *s*^*37C*^, are (Atkins and de Paula, 2006)

(38) $$h_{f}^{37C}=h_{f}^{o}+c_{p}\left(310.15\;K-298.15\;K\right)=h_{f}^{o}+h_{cor}$$

(39) $$s^{37C}=s^{o}+c_{p}\;\ln\frac{310.15\;K}{298.15\;K}=s^{o}+s_{cor}$$

where *c*_*p*_ is specific heat capacity, while *h*_*cor*_ and *s*_*cor*_ are corrections that need to be made from 298.15K to 310.15K. The specific heat capacity of dry yeast biomass is 1.308 J/g K (Battley et al., 1997). Based on these values, *h*_*cor*_ = 0.0157 kJ/g and *s*_*cor*_ = 0.0516 J/g K. Both of these corrections are lower than the error values reported in Table 2 (0.27 and 0.26, for yeast *h*_*f*_*⁰* and *s*⁰, respectively). Thus, for calculations at 37 °C it is an acceptable approximation to use the standard thermodynamic parameter values reported in Tables 1 and 2.

## 4 Discussion

This section begins with a discussion of the general trends in the results from section 3 and attempts to give explanations for these trends. Then microorganism metabolic rates are discussed. Finally, a thermodynamic analysis of microorganism growth is made. The analysis first determines entropy of individual E. coli and *Pseudomonas* cells, and then entropy of a *Pseudomonas* colony during its lifespan.

### 4.1 Trends in microorganism thermodynamic parameters

Most living cells constitute of similar classes of molecules and thus have similar thermodynamic properties. All the analyzed microorganisms have very similar *(h*_*f*_*⁰)*_*bio*_, *s*⁰_*bio*_ and *(g*_*f*_*⁰)*_*bio*_ values, respectively (Fig. 1). The reason is that most living organisms share a very similar molecular structure: they are made mostly of water, lipids, proteins, carbohydrates and nucleic acids (Alberts et al., 2002). These compounds are major components of all living cells, because all existing life forms have evolved from a single common ancestor (last universal common ancestor, LUCA). The differences in cell phenotypes arise from the expressed proteins and distribution of constituent molecules. However, as can be seen from the general empirical formulas of bacteria (CH_1.7_O_0.4_N_0.2_), fungi (CH_1.7_O_0.5_N_0.1_) and algae (CH_1.7_O_0.5_N_0.1_), the general chemical constituents of all the three groups of organisms are very similar, resulting in similar elemental compositions and similar values of *(h*_*f*_*⁰)*_*bio*_, *s*⁰_*bio*_ and *(g*_*f*_*⁰)*_*bio*_.

Formation of biomass from elements is always exothermic because there is partial oxidation of less electronegative elements in the biomass by oxygen and nitrogen. All analyzed microorganism species have very similar *(h*_*f*_*⁰)*_*bio*_ which are all negative. The reason can be seen from the chemical equation representing biomass formation from elements:

(40) n_c_ C + ½ n_H_ H_2_ + ½ n_O_ O_2_ + ½ n_N_ N_2_ + n_P_ P + n_S_ S + n_K_ K + n_Mg_ Mg + n_Ca_ Ca + n_Fe_ Fe → C_nC_H_nH_O_nO_N_nN_P_nP_S_nS_K_nK_Mg_nMg_Ca_nCa_Fe_nFe_

Oxygen is the second most electronegative atom in the periodic table, after fluorine. Since there are no oxygen-fluorine compounds in the biomass and peroxides are not present in a high concentration, the oxidation state of oxygen is -2. Similarly, nitrogen is in the biomass mostly present in the form of amines, making its oxidation state -3. Thus, during formation of biomass from elements, oxygen and nitrogen change their oxidation states from 0 in their elemental forms to -2 and -3, respectively, in the biomass. This implies that there is partial oxidation of the other elements that constitute the biomass. This partial oxidation process, like all other oxidations, is exothermic and makes the enthalpy of formation of biomass from elements negative. This is in agreement with the results of Erickson et al. (1978, p. 1598), who found that microbial biomass synthesis from organic substrates (such as glucose) is exothermic, due to partial oxidation of the substrate by oxygen.

### 4.2 Microorganism metabolic rates

Metabolic rate of a living organism is the amount of energy it uses to sustain itself for a period of time and is expressed in watts. Specific metabolic rate is the metabolic rate of an organism divided by its mass and is expressed in watts per kilogram of body weight. For microorganisms, there are two metabolic rates of interest: endogenous metabolic rate and growth metabolic rate (Makarieva et al., 2005). Endogenous metabolic rate is the metabolic rate of nongrowing unicellular organisms in nutrient-free suspensions (Makarieva et al., 2008). It is the microorganism analog of basal or standard metabolic rate in animals (Makarieva et al., 2008). On the other hand, growth metabolic rate is the metabolic rate of microorganisms growing on a medium (Makarieva et al., 2005). Specific endogenous metabolic rates of some of the microorganisms in this study are given in Table 6.

**Table 6:** Specific endogenous metabolic rates of microorganisms. The data was taken from Makarieva *et al.* (2008).

Name	q (W/kg-wet mass)	m_cell_ (pg)
Aerobacter aerogenes	8.71	0.4
Bacillus cereus	21.92	3.7
Escherichia coli	5.22	0.7
Escherichia coli K-12	14	0.7
Escherichia coli W	4.2	0.7
Klebsiella aerogenes	7.07	0.3
Paracoccus denitrificans	5.87	0.16
Chlamydomonas reinhardtii	15	19.4
Chlorella pyrenoidosa	7.7	30
Selenastrum capricornutum	21	22.432

Basal metabolic rates of many organisms can be predicted using Kleiber's law, which states that basal metabolic rate of an organism *Q* is proportional to its mass *m*: *Q* = 293 *m*^*3/4*^ (Kleiber, 1947; Balmer, 2010). This implies that the specific basal metabolic rate *q* is *q* = 293 *m*^*−1/4*^ (Balmer, 2010). Kleiber's law has been found to give good predictions of metabolic rates of animals (Balmer, 2010). However, Hemmingsen found that Kleiber's law does not hold for microorganisms (Blaxter, 1989, page 126). This result was confirmed in a later study by Makarieva et al. (2005), which involved 80 prokaryote species, and by Makarieva et al. (2008), involving 3006 species from all kingdoms. Thus, for microorganisms it is best to use their experimental metabolic rates. A comprehensive database with basal metabolic rates of many living organisms, including endogenous metabolic rates of microorganisms, can be found in the supplemental material of Makarieva et al. (2008). Growth metabolic rates of microorganisms are given in the supplemental material of Makarieva et al. (2005).

### 4.3 Thermodynamic analysis of microorganism growth

In this section, the results from section 3 will be applied to bacterial colony growth. Entropy of a single E. coli cell and a single *Pseucomonas* cell will be calculated, and entropy change of a growing *Pseudomonas* colony will be analyzed. The following analysis is based on two axioms:

1. Microorganisms grow and multiply, making colonies, through cell division.
2. Growth is caused by import and accumulation of substances taken from the surroundings, resulting in change in mass and volume of a colony.

Growth of microorganisms occurs in five phases, during time and culture aging: (1) lag, (2) exponential, (3) declining growth rate, (4) stationary and (5) death phase. Duration of the phases depend on availability of nutrients in the environment. The exponential phase is the period when growth is the most intense. The number of microorganisms is described as a function of time by the equation

(41) $$N_{cells}=N_{cells,0}\;2^{t/t_{d}}$$

where *N*_*cells,0*_ is the initial cell number and *t*_*d*_ is division time (Widdel, 2010). A cell or a colony, as growing open systems, are characterized by thermodynamic parameters, including entropy (Battley, 1999a; von Stockar and Liu, 1999).

Entropy of a single E. coli cell was calculated using data from Table 2. A cell consists of dry biomass and water. Therefore, the total entropy of a cell, *S*_*cell*_, is

(42) $$S_{cell}=m_{bio}\cdot s_{bio}^{o}+\;m_{w}\cdot s_{w}^{o}+{\left(S_{P}\right)}_{hyd}$$

where *s*⁰_*bio*_ is standard specific entropy of dry biomass (Table 2), *m*_*bio*_ mass of dry biomass, *m*_*w*_ mass of water in the cell, and *s*_*w*_⁰ standard specific entropy of water, which is 3.886 J/g K (Cox et al., 1984). Eq. (42) also contains entropy of hydration of biomass, *(S*_*P*_*)*_*hid*_. However, there is no method to accurately predict *(S*_*P*_*)*_*hid*_. Thus, *(S*_*P*_*)*_*hid*_ was included into the error of *s*⁰_*bio*_ given in Table 2, as is described in section 2.3, and *(S*_*P*_*)*_*hid*_ was set to zero in Eq. (42). Finally, a single Escherichia coli cell weighs 9.5 · 10^−13^ g, containing 2.8 · 10^−13^ g (30%) of dry mass and 6.7 · 10^−13^ g (70%) of water (Neidhardt, 1996). Therefore, according to Eq. (42) the entropy of a single E. coli cell is (3.01 ± 0.05) · 10^−12^ J/K. A similar reasoning can be applied to a *Pseudomonas* cell. A *Pseudomonas* cell has a mass of 8 · 10^−13^ g, of which 70% is water (Makarieva et al., 2008). Using data from Table 2, entropy of a single *Pseudomonas* cell is (2.55 ± 0.04) · 10^−12^ J/K.

**Fig. 2:**
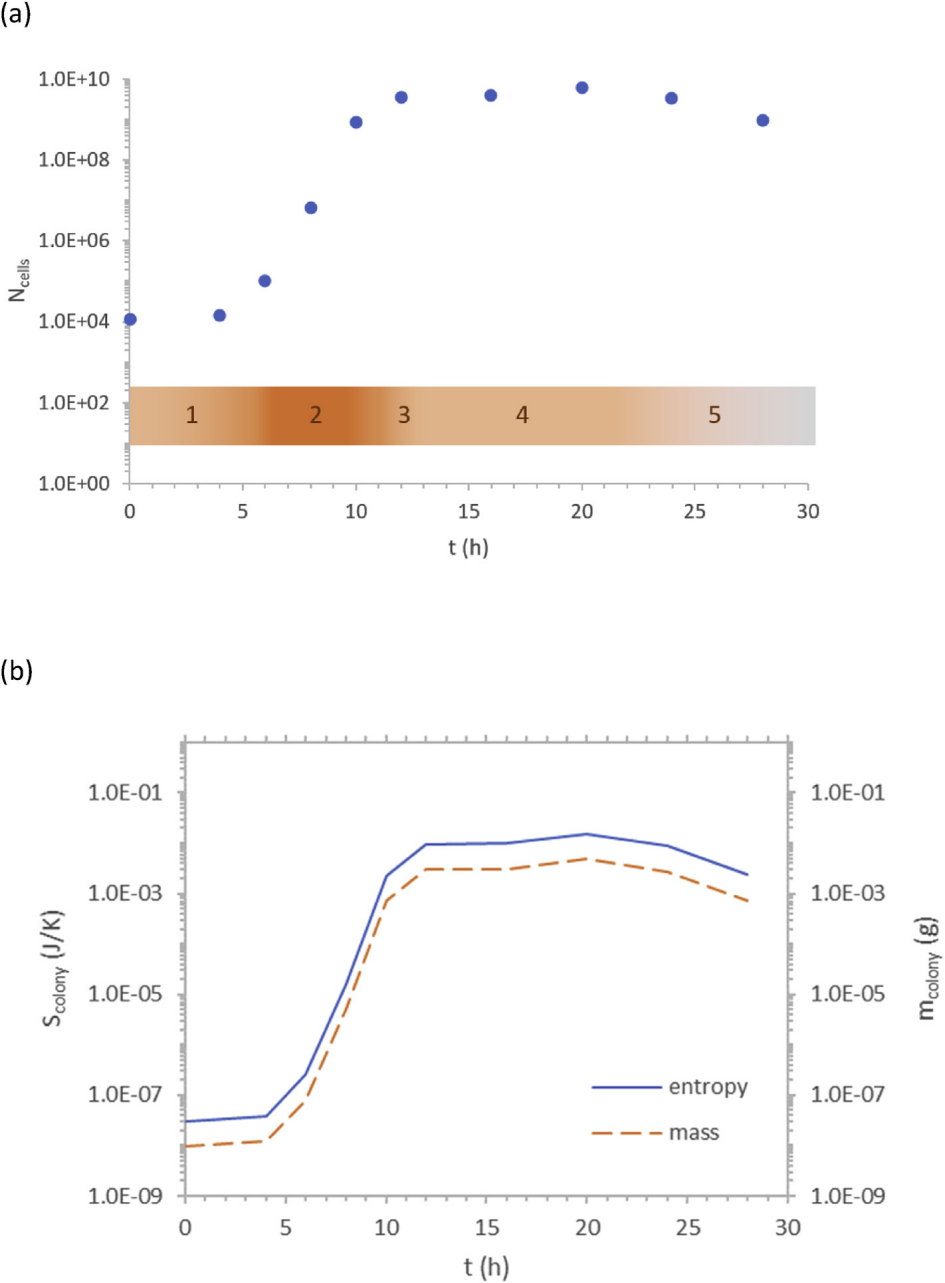
Thermodynamics of growth of a *Pseudomonas* colony. (a) Cell number versus time - growth curve data taken from Maier et al. (2009). The orange gradient line represents growth intensity, while the numbers on it indicate the growth phases: (1) lag, (2) exponential, (3) declining growth rate, (4) stationary and (5) death phase. (b) Colony entropy *S*_*colony*_ and mass *m*_*colony*_ as a function of time. Colony entropy was calculated through Eq. (43), using the previously calculated single cell entropy of (2.55 ± 0.04) ∙ 10^−12^ J/K. Colony mass was calculated as cell mass multiplied by number of cells. The entropy curve is represented by the full blue line, while the dashed orange line represents the mass curve.

Based on entropy of a single cell and bacterial growth data, entropy of an E. coli colony was calculated as a function of time. Microorganisms live in colonies. The entropy of a colony is the sum of entropies of all microorganisms that comprise it

(43) $$S_{colony}=N_{cells}\text{·}S_{cell}$$

where *N*_*cells*_ is the initial number of cells in the colony. Since a colony grows during time through increase in cell number, its entropy changes. Therefore, using Eq. (43) and growth data, entropy of a microorganism colony can be determined as a function of time. Using growth data from Maier et al. (2009), entropy of a *Pseudomonas* colony throughout its lifespan was calculated and is shown in Fig. 2.

As can be seen from Fig. 2, there is an exponential increase in both the number of cells and in entropy of the colony during the exponential phase of colony growth. This is a general trend, according to Eq. (43), since standard specific entropy of any substance, including living organisms, can only be positive due to the third law of thermodynamics, as discussed in section 3. Therefore, any growing organism increases its mass and entropy.

Three periods can be distinguished in the existence of a microorganism colony: (a) accumulation period when cell number, mass and entropy increase, (b) steady state period when they are approximately constant, and (c) decumulation period when they decrease. The *Pseudomonas* colony begins growth with 1.2 · 10^4^ cells and an initial entropy of 3.1 · 10^−8^ J/K. Then, through import, accumulation of matter from the environment and cell division, the colony grows, and increases its mass and entropy to 9.4 · 10^−3^ J/K, after 12 hours. It is obvious that entropy of microorganisms, during colony life and aging, increases during the first three phases (lag, exponential and declining growth rate), corresponding to the accumulation period. During the fourth phase (stationary), cell number and entropy remain approximately constant in time, making the steady state period. Only in the last phase (death), due to lack of nutrients, the entropy of the colony begins to decrease due loss of living cells and the colony enters the death period. Finally, once the last cell has decomposed, the colony ceases to exist.

## 5 Conclusions

Elemental compositions have been collected and presented for 32 microorganism species, including 14 bacteria, 7 yeast and 11 algae species (Tables 1 and 2). All three classes have very similar average elemental compositions: bacteria CH_1.7_O_0.4_N_0.2_, fungi CH_1.7_O_0.5_N_0.1_ and algae CH_1.7_O_0.5_N_0.1_.

Enthalpy of combustion experimental data was used to compare five widely used predictive models: Patel-Erickson, Boie, Dulong, Mason-Gandhi and Channiwala-Parikh equations (Table 5). It was found that Patel-Erickson and Channiwala-Parikh equations give the most accurate predictions for microorganism biomass. The Patel-Erickson model was chosen to perform the calculations in this work, since it is the most appropriate for microorganism biomass composition data. Based on standard enthalpies of combustion of microorganism biomass, standard enthalpies of formation from elements were calculated.

Standard entropy of biomass was found from its elemental composition using the Battley equation, a model that can give accurate predictions for a wide range of organic compounds. Based on standard enthalpy of formation from elements and entropy, standard Gibbs free energy of formation from elements was calculated. Thermodynamic properties were calculated for the 32 microorganism species (Tables 1 and 2).

Trends in microorganism thermodynamic parameters were discussed. Each thermodynamic property has very similar values for all the analyzed microorganisms (Fig. 1). This is due to the fact that all living organisms share a universal common ancestor and thus have similar chemical compositions. Standard enthalpies of formation from elements of all analyzed microorganisms are negative, due to the fact that during hypothetical formation of biomass from elements oxygen and nitrogen partially oxidize other elements in the biomass. All microorganisms have positive standard specific entropy, due to the third law of thermodynamics. Gibbs energies of formation from elements of all the microorganisms are negative.

A brief review was made of microorganism endogenous and growth metabolic rates (Table 6). It has been found that it is best to use experimental values, since the subject literature shows that microorganism metabolic rates cannot be correlated using Kleiber's law.

Entropy a single *E.* *coli* cell has been found to be (3.01 ± 0.05) · 10^−12^ J/K, while entropy of a single *Pseudomonas* cell is (2.55 ± 0.04) · 10^−12^ J/K. Based on this value, entropy of a *Pseudomonas* colony was calculated during its lifespan (Fig. 2). During the log, exponential and declining growth rate phases entropy and mass of the colony both increase, in the stationary phase they are approximately constant, while in the death phase they decrease. Thus, three periods can be distinguished in the existence of a microorganism colony: accumulation period, steady state period, and decumulation period.

## Declarations

### Author contribution statement

Marko Popovic: Conceived and designed the experiments; Performed the experiments; Analyzed and interpreted the data; Contributed reagents, materials, analysis tools or data; Wrote the paper.

### Funding statement

This research did not receive any specific grant from funding agencies in the public, commercial, or not-for-profit sectors.

### Competing interest statement

The authors declare no conflict of interest.

### Additional information

No additional information is available for this paper.
